# Standards for the Reporting of Genetic Counseling Interventions in Research and Other Studies (GCIRS): an NSGC Task Force Report

**DOI:** 10.1007/s10897-017-0076-9

**Published:** 2017-02-24

**Authors:** Gillian W. Hooker, D Babu, MF Myers, H Zierhut, M McAllister

**Affiliations:** 1 NextGxDx, 810 Crescent Centre Dr, Suite 280, Franklin, TN 37067 USA; 2 Ambry Genetics, Aliso Viejo, CA USA; 3 Cincinnati Children’s Hospital Medical Center and University of Cincinnati, Cincinnati, OH USA; 4 University of Minnesota, Minneapolis, MN USA; 5 Cardiff University, Cardiff, UK

**Keywords:** Reporting standards, Genetic counseling research, Genetic counseling intervention

## Abstract

As the demand for evidence to support the value of genetic counseling increases, it is critical that reporting of genetic counseling interventions in research and other types of studies (e.g. process improvement or service evaluation studies) adopt greater rigor. As in other areas of healthcare, the appraisal, synthesis, and translation of research findings into genetic counseling practice are likely to be improved if clear specifications of genetic counseling interventions are reported when studies involving genetic counseling are published. To help improve reporting practices, the National Society of Genetic Counselors (NSGC) convened a task force in 2015 to develop consensus standards for the reporting of genetic counseling interventions. Following review by the NSGC Board of Directors, the NSGC Practice Guidelines Committee and the editorial board of the *Journal of Genetic Counseling*, 23 items across 8 domains were proposed as standards for the reporting of genetic counseling interventions in the published literature (GCIRS: Genetic Counseling Intervention Reporting Standards). The authors recommend adoption of these standards by authors and journals when reporting studies involving genetic counseling interventions.

## Introduction

Increasingly, as methodologies for synthesizing published health research mature (Moher 2009), reporting standards have been developed to allow for critical assessment of methodological quality, interpretation of findings, and comparison of findings across studies. Reporting standards, often presented to authors in checklist format, are guidelines intended to promote accurate and complete reporting of research by outlining a core set of components to be reported at the time of publication. Some currently used reporting standards include the CONSORT (Moher et al. 2012) for randomized controlled trials, the TIDieR (Hoffmann et al. 2014) for a broad range of interventions, and, in genetics, the STREGA (Little et al. 2009) for genetic association studies. These standards are used by authors to guide the reporting of specific methods, interventions or findings and are commonly included in the instructions to authors provided by journals. Where checklists are used, they may be intended as guides for authors only, or they may be required by journals to accompany submissions.

Standards like these stand to bring clarity and improvement to the reporting of genetic counseling intervention studies. The CONSORT standards for reporting of randomized trials are useful to support overall study design and reporting of randomized trials of genetic counseling interventions, but they lack sufficient detail to enable replication of interventions (Hoffmann et al. 2014). The TIDieR standards were developed to fill this gap, and are generally applicable to all intervention studies.

A health “intervention” is any activity undertaken to prevent, improve, or stabilize a medical condition. Arguably, genetic counseling is a “complex intervention” [i.e. a multi-dimensional intervention with several interacting components (Campbell et al. 2000; Craig et al. 2008)]. In the case of genetic counseling, the intervention can comprise counseling, risk assessment, education and risk communication. Furthermore, genetic counseling may itself be one part of an even larger complex intervention, comprising additional components (e.g. genetic testing and physical examination). Complex interventions present special problems for the evaluators of the intervention, not least of which is how to standardize the design and delivery of the intervention. In the past, heterogeneity in reporting of key components of genetic counseling (e.g., education and psychosocial elements of the intervention, background and training of those providing genetic counseling, and mode of delivery of the intervention) has led to ambiguity in secondary analyses of the published literature and hindered the translation of research findings into evidence-based practice (Heshka et al. 2008).

Differing opinions about the definition and goals of genetic counseling add further complexity to the interpretation and reporting of studies involving genetic counseling (Ormond 2013; Resta et al. 2006). Comprehensive specification of (novel) genetic counseling interventions in research and other publications will enable interpretation of the value of the intervention, including patient benefits. Accurate translation and replication of study findings by others will contribute to implementation of evidence-based clinical applications in genetic counseling.

### Scope of the GCIRS

The intent of the GCIRS (Genetic Counseling Intervention Reporting Standards) is to outline a set of standards for the specific reporting of genetic counseling interventions in research and other publications (e.g. service evaluation and quality improvement studies). This document is likely to be most beneficial for the reporting of genetic counseling interventions performed with the intent of evaluating genetic counseling outcomes (e.g. in randomized controlled trials supporting clear specification of both novel interventions and standard care “control” interventions, so that the contribution of the novel intervention over standard care is clear). It is conceivable that in some studies, such as those that compare genetic counseling with and without genetic testing, the novel and standard care genetic counseling interventions might be identical. However, clear specification of the genetic counseling intervention will still contribute significantly to enabling study replication. While these standards are intended to guide reporting, they may also be useful to those designing genetic counseling studies. In addition, these standards are not intended to replace other research reporting standards (e.g. CONSORT, TIDieR), but to be used alongside these other standards to support more accurate specification of genetic counseling interventions. Further, they are not intended to be prescriptive for genetic counseling practice. The implementation of these standards should facilitate more consistent and rigorous synthesis and translation of the literature comprising the evidence base for genetic counseling. As researchers gain experience with these standards and methodological evidence accrues, updates may be needed to these standards.

## Methods

A task force of five experts in genetic counseling practice and/or research was appointed by the NSGC Board of Directors to represent different stakeholder groups from within the NSGC and to author the standards. The authors were selected based on their experiences conducting genetic counseling research, editorial board service for the *Journal of Genetic Counseling*, and/or service on various NSGC committees such as the Practice Guidelines Committee and Jane Engelberg Memorial Fellowship Advisory Board. All authors are genetic counselors who have practiced clinically, with years of clinical experience ranging from 5 to 15 years. Four of the five have additional training in social research, epidemiology and health services research. All authors have experience with qualitative and quantitative research methodologies. Three authors are U.S.-based, one is in Canada and one in the United Kingdom.

The group began by looking at studies included in a systematic review completed as part of the NSGC Outcomes Work Group (Madlensky et al. 2017). Studies included in the systematic review included pre-post genetic counseling designs and intervention studies comparing a genetic counseling arm with some other intervention or a control arm that did not include genetic counseling (case-control design). It was evident that many of the studies included in the review did not specify genetic counseling interventions in a way that enabled clear replication of the intervention. All five members independently assembled a list of domains and items appropriate for genetic counseling reporting standards, and then reconvened to discuss them. The domains and items were iteratively compiled to remove redundancy as well as any elements considered outside the scope of the standards. Discrepant items were discussed and resolved by group consensus., also in an iterative manner, by e-mail and telephone. All members then reviewed and edited the draft list of domains and items and later compared them with the TIDieR standards for reporting of interventions. There was consensus amongst the author group that the genetic counseling community would benefit from supplementation of standards like TIDieR to support clear specification of genetic counseling interventions. Once an initial consensus was reached, the NSGC Board of Directors, the NSGC Practice Guideline Committee and, as an end-user quality measure, the editorial board of the *Journal of Genetic Counseling,* reviewed the final list of domains and items. Comments and feedback from these groups were incorporated to refine and revise the domains and supporting documentation.

The second author piloted the original checklist during review of an article describing a genetic counseling intervention. The pilot exercise revealed that: (1) overall, the checklist was easy to use, (2) the domains were easy to understand and identify when reviewing, and (3) many of the domains in the manuscript under review were missing or not well described, suggesting that the tool will be useful for reviewers and will also assist those writing grants and designing and reporting studies. The pilot activity also identified the need for study authors to describe the difference between two or more interventions (e.g. novel versus standard care), so this clarification was added to the checklist.

The pilot activity and the review of the original standards indicated some confusion about certain variables in the standards. An original domain name “Clinical context” was particularly unclear and did not appear to fully capture the intended content of the domain. This led to changing the title of the domain “Clinical context” to “Other components of a complex intervention” and adding further explanation of the domain. In addition, in response to reviewer comments, we added a “Not applicable” column to the checklist and more clearly separated domains and sub-domains in the checklist table. We completed this entire process between June of 2015 and July 2016.

## Results

The task force produced a final checklist of 23 items classified within 8 domains (see Table 1). This list is designed for reporting across a range of different study designs that include genetic counseling. In some cases, specific items may not be applicable. For example, some studies of genetic counseling may be conducted in settings where genetic testing is not commonly offered or available. Alternatively, other genetic counseling interventions may be focused on a specific educational (e.g., recall of risk information) or counseling goal, and therefore may not include other genetic counseling components. If the study includes a comparison or control group, the domains and items should be described for both groups, as applicable. Explanations for each domain and the items within it are provided below.

**Table 1:** Checklist for reporting genetic counseling interventions*

Domain	Item	Reported on page no.	Not Applicable (NA) and/or Not Recorded (NR)
1. Indication for genetic counseling	1. Reason for genetic counseling intervention	—	—
	2. Affected status of the counselee (clinically symptomatic/asymptomatic) at time of genetic counseling intervention	—	—
2. Other components of a complex intervention	3. Other evaluations at the time of genetic counseling intervention (e.g. other clinician interactions, physical examination)	—	—
	4. Genetic testing before, after, or at the time of counseling	—	—
	5. Testing indications or risk thresholds for testing	—	—
3. Intervention delivery	6. Delivery mode (telephone, in person, telemedicine, video; with interpreter; individual, couple or group; if group, group size and composition)	—	—
	7. Physical setting (hospital, clinic, public vs. private)	—	—
	8. Payment method (genetic counseling paid for by participant, private or government insurance, or grant funding, whether participants received incentive)	—	—
4. Provider(s) of genetic counseling	9. Qualifications or other training/credentials	—	—
	10. Number and type(s) of healthcare professionals involved in the genetic counseling interaction with each participant	—	—
	11. Number and type(s) of healthcare professionals delivering intervention in study	—	—
5. Risk content and communication	12. Basis of risk assessment (family history, test results, personal history, tools and algorithms)	—	—
	13. Type and format of risk information provided to participant. (e.g. frequencies, odds ratios, absolute/relative risk etc.)	—	—
6. Educational content	14. Educational goals and learning objectives of genetic counseling intervention	—	—
	15. Educational tools employed (visual aids, interactive web applications, other)	—	—
	16. Educational models or theories applied	—	—
7. Psychotherapeutic content	17. Psychotherapeutic goals (e.g decision making, promoting family communication, facilitating coping and adaptation)	—	—
	18. Psychotherapeutic tools and techniques employed	—	—
	19. Psychotherapeutic models or theories	—	—
8. Duration	20. Length of each genetic counseling interaction	—	—
	21. Number of genetic counseling interactions	—	—
	22. Time between genetic counseling interactions	—	—
	23. Follow-up genetic counseling interactions	—	—

*It is intended that a separate checklist be completed for each genetic counseling intervention arm included in a study, so that any differences between a novel intervention and standard care are clear

### Domain 1: Indication

This domain provides background information regarding why a genetic counseling intervention was performed for the study population. Items in this domain may include the study participants’ demographics (e.g., age, gender, ethnicity), medical history (e.g., personal genetic test results, whether they were symptomatic or asymptomatic, other relevant diagnoses or biomarkers, age of onset, time since any diagnoses) or family history. This domain may also cover broader, population-level indications for genetic counseling interventions, such as antenatal or pre-conception screening or genetic counseling interventions for common diseases.

### Domain 2: Other Components of a Complex Intervention

The items in this domain are intended to capture whether any other interventions occurred at the time of genetic counseling (e.g., physical examinations, other medical (non-genetic) tests, other clinician interactions), and whether genetic testing was offered or ordered prior to, at the time of, or following genetic counseling. Of key importance is whether genetic counseling was performed in isolation or whether it was provided as part of a complex intervention comprising other interventions to which outcomes could be attributed. Precise details of all other appointments and evaluations may not be required.

### Domain 3: Intervention Delivery

Included in the intervention delivery domain are items intended to capture the method by which the genetic counseling intervention was delivered to the clients. Specifically, the authors should describe whether genetic counseling was delivered in person or using an alternative mode (e.g. video, telephone, live teleconference, webinar), whether it was delivered to an individual or group, whether it was delivered through an interpreter for some or all participants along with any credentials or certifications held by that interpreter; and where the study participants were physically located during the session (e.g. hospital, community health center, public vs. private clinic). If genetic counseling was provided to a group, the size and composition of the group should also be reported. Included in this domain is an item to describe if and how genetic counseling was paid for (by insurance, out-of-pocket, government insurance, through research funding) and whether participants were provided financial or other incentives to participate.

### Domain 4: Provider(s)

The qualifications, training, credentials (e.g. certification, registration) and clinical experience of the individuals providing the genetic counseling intervention should also be reported. Further, recognizing that many teams or clinics are multidisciplinary, the number of providers seen by each participant at the time of their research visit should be reported, along with the total number of healthcare professionals providing the genetic counseling intervention, both to an individual participant and across the study as a whole.

### Domain 5: Risk Content and Communication

Core components of many genetic counseling interventions are risk assessment through the collection of family history data or other risk assessment tool(s) or algorithms, and communication of this information to guide discussions about risk. This domain guides authors to report whether and how risk was assessed, including whether family history information was collected and whether a pedigree was constructed, any pedigree conventions employed and any other tools used to generate risk figures. The way risk information was communicated to participants should be described: whether a qualitative or quantitative probability of disease was communicated, and if quantitative, the type and format of risk presentation. The basis for the risk estimate should also be reported (e.g. family history, genetic testing, online risk assessment tool, personal history, empiric population risks).

### Domain 6: Educational Content

For any educational content included in the genetic counseling intervention, authors should describe what the goals or learning objectives of the intervention were, what the methods or tools used to achieve these goals were, the content included in the educational component, and what, if any, educational theories or models guided their approach. Examples of methods or tools might be visual aids, interactive learning exercises, and videos or other multi-media.

### Domain 7: Psychotherapeutic Content

The psychotherapeutic goals and objectives of the genetic counseling intervention should also be specified, as well as the counseling approaches and interventions used to address those goals and their theoretical underpinnings. Counseling goals might include facilitating decision-making and/or informed consent, reducing psychological distress, promoting feelings of personal control and empowerment, facilitating family communication, and supporting adaptation to stressful life events. Examples of counseling interventions might be active listening, motivational interviewing, confrontation, reflection, and role-playing. Examples of theories or models authors might specify include cognitive behavior theory, family systems theory and stress and coping models.

### Domain 8: Duration

The length and number of the genetic counseling intervention(s) should be reported, as well as the time interval between consultations if applicable. For example, some studies may include pre- and post-test counseling, in which case the length of each session and the time between the sessions should be reported. Any follow-up constituting additional communication between the person providing the genetic counseling and the participant within the study period (phone calls, letters, follow-up meetings) should also be reported.

## Conclusions and Implementation

The GCIRS checklist is intended as a tool for authors reporting studies of genetic counseling interventions to promote synthesis and translation of research and other findings into genetic counseling practice. These standards are not intended to dictate genetic counseling practice, study design, or conduct of genetic counseling research, but are intended to support more accurate reporting of genetic counseling interventions when publishing. Standards for reporting research and other types of studies bring additional transparency to the process and facilitate consistent interpretation and comparison of findings across investigations. Reporting standards may also lead to accurate replication of studies and clearer understanding of how to build upon previous findings. Finally, such standards may ultimately lead to integration of novel genetic counseling interventions into evidence-based clinical practice.

The domains included in the checklist reported here overlap significantly with the general domains included in the TIDieR standards, intended to support intervention description and replication (Hoffmann et al. 2014). For example, the TIDieR standards recommend reporting intervention provider (TIDieR item 5), mode of delivery (TIDieR item 6), intervention location (TIDieR item 7) and when and how much (TIDieR item 8), all of which are included in Table 1 here. The GCIRS standards add value for genetic counseling interventions in the more specific domains of “Indication,” “Other components of a complex intervention,” “Risk content and communication,” “Educational content” and “Psychotherapeutic content.” The GCIRS standards can be used in conjunction with the TIDieR standards for reporting of studies that include one or more genetic counseling interventions. They can also be used in conjunction with the CONSORT standards for reporting of randomized trials that include one or more genetic counseling interventions. For instance, the CONSORT checklist specifies reporting for details about participant recruitment and randomization (i.e. participant flow and fidelity) not included in GCIRS (Moher et al. 2012). The GCIRS checklist includes a number of items specific to a genetic counseling intervention, not included in CONSORT, to further delineate how an intervention could be repeated in the context of genetic counseling.

The details outlined in the reporting standards should typically be described in the Methods section of articles reporting research involving genetic counseling interventions. However, if there are instances where they reflect data collected within the study, they may be more appropriately reported in the Results section (e.g. average duration between counseling sessions). The checklist may be used by authors to support their submissions and could also be used by journals to standardize reporting in the papers they publish. Some items in the checklist may not apply to a particular study; in this case, authors could indicate that these items are not applicable to their study or, in some cases, not recorded as a part of the study. Further, the examples provided in this document are not comprehensive or intended to clarify specific components of the standards, and do not indicate preferred or accepted models of practice. Rather, they are intended clarify by illustrating specific components of the standards. Other standards documents such as this one have brought considerable rigor to other fields of research. The CONSORT guidelines have been cited in thousands of studies, endorsed by over 600 journals and have significantly improved the reporting of randomized controlled trials (Turner et al. 2011).

Improved reporting of genetic counseling interventions provided in a research context may help advance key initiatives of critical importance to the growth and development of the field and delivery of high-quality service. As the field of genetic counseling moves toward evidence-based practice to increase the likelihood of achieving desirable patient and system-level outcomes, well-synthesized, standardized evidence will be required to develop strong clinical practice guidelines. The NSGC Practice Guidelines process, in keeping with national standards for trustworthy clinical practice guidelines (Institute of Medicine 2011), requires systematic reviews of the evidence, which in turn are dependent upon synthesizable evidence.

It will likely be necessary to update these standards every five years to meet the changing healthcare needs related to genetic counseling and to address any methodological oversights that come to light once they begin to be applied in practice. Given the parallels between the GCIRS standards and the TIDieR standards (the latter developed following a Delphi exercise) and recognizing the importance of community engagement in standards development (Moher et al. 2010), we strongly recommend that the research community work collaboratively to collect and report data regarding the use and impact of the standards to inform revisions to and further strengthen the GCIRS. If these standards are, through use, found to be valid and to meet their intended purpose, the overall quality of reporting of genetic counseling interventions in research and other types of studies should improve. Secondarily, the ability of researchers to conduct and interpret secondary research (meta-analyses, systematic reviews) should improve. The overall goal of these standards is to facilitate systematic reporting of genetic counseling research and genetic counseling interventions. To that end, we encourage authors publishing such research to use these standards in future publications.
